# Understanding low-threshold mode-locking at multi-GHz repetition rate

**DOI:** 10.1038/s41377-024-01682-0

**Published:** 2025-01-02

**Authors:** Wenbin He, Meng Pang, Philip St. J. Russell

**Affiliations:** 1 Russell Centre for Advanced Lightwave Science, Shanghai Institute of Optics and Fine Mechanics and Hangzhou Institute of Optics and Fine Mechanics, Chinese Academy of Sciences, Shanghai, 201800 China; 2 State Key Laboratory of High Field Laser Physics and CAS Center for Excellence in Ultra-intense Laser Science, Shanghai Institute of Optics and Fine Mechanics CAS, Shanghai, 201800 China

**Keywords:** Mode-locked lasers, Ultrafast lasers

## Abstract

Continuous-wave mode-locking at multi-GHz repetition rates is achieved in an ultrashort laser cavity at critical pulse energies 100 times lower than predicted by conventional theory. The authors reveal that dynamic gain depletion and recovery between consecutive round-trips is the key factor behind a low-pulse-energy transition from Q-switched mode-locking (QSML) to continuous-wave mode-locking (CWML). As well as providing new insight into gain dynamics, the results suggest a practical route to low-threshold lasing at very high-repetition rates.

Laser mode-locking using saturable absorbers provides a simple and robust method for generating trains of ultrashort pulses^1^. Commonly used in many applications and much studied, mode-locked lasers are intrinsically complex systems with a range of different operating regimes^2^. The authors identify a new regime that favours multi-GHz CWML over QSML at very low critical pulse energy.

Q-switched bursts of mode-locked pulses are governed by relaxation oscillations, and occur when the individual pulse energy is lower than a critical value determined by the laser configuration and varies not only over each cavity round-trip due to gain/loss competition, but also over many round-trips. Conventional theory predicts that the critical pulse energy for the onset of CWML and suppression of QSML is determined by gain saturation and the saturable absorber threshold^3^. The gain saturation must be sufficient to damp out any relaxation oscillations, and the pulses strong enough to bleach the saturable absorber.

Taking a fresh look at high-repetition-rate CWML in a soliton fibre laser with an ultrashort cavity and a semiconductor saturable absorber mirror (SESAM)^4^, Chen et al. have found, unexpectedly, that QSML can be suppressed at pulse energies two orders of magnitude lower than predicted by conventional theory. They achieve stable 21 GHz CWML at a critical pulse energy of only ~24 pJ. Although there are previous reports of critical energies that are puzzlingly much lower than theoretical predictions, this is the first time these have been systematically explained.

Chen et al. developed a new theory by considering dynamic gain depletion between consecutive round-trips. Conventionally the gain is assumed to be saturated and time-independent, fully recovering after each round-trip (Fig. 1a). While this is valid at low enough repetition rates (kHz to MHz), at multi-GHz rates the pulse spacing is too short for full gain recovery so that the gain can no longer be treated as time-independent, with the result that successive pulses become correlated by gain depletion. If the pump power is too low, the dynamic gain drops after each round-trip due to gain depletion, until solitons are no longer supported; the gain then fully recovers. The result is quasi-rectangular bursts of QSML pulses as observed in the experiments, reminiscent of soliton crystals^5^ (Fig. 1b). Note that in such states successive solitons within a single burst will have slightly lower energies. As the pump power increases, these “soliton crystals” merge to form an unbroken CWML pattern.

**Fig. 1:**
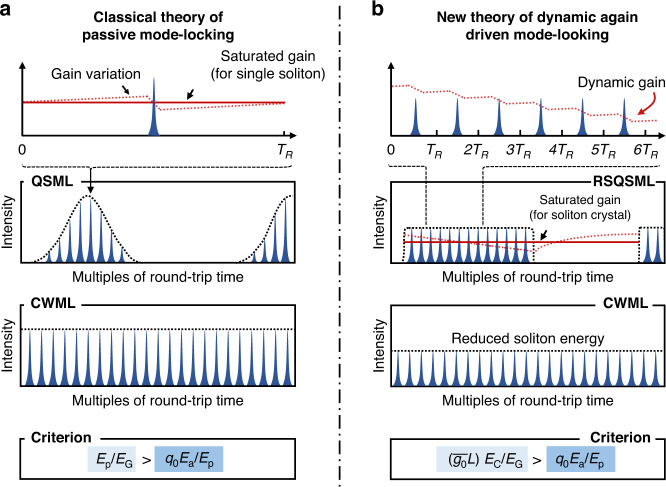
Descriptions of gain evolution (top), Q-switched and stable mode-locked states (middle), and criteria (bottom) in the classical theory of mode-locking (**a**) and new theory of dynamic gain driven mode-locking (**b**). QSML, Q-switched mode-locking. CWML, continuous-wave mode-locking. RSQSML, rectangular-shape QSML. ${E}_{{\rm{p}}}$ is the soliton energy.$\,{E}_{{\rm{G}}}$ and ${E}_{{\rm{a}}}$ are the saturation energy of the gain medium and absorber. ${q}_{0}$ is the modulation depth of the absorber. $\bar{{g}_{0}}$ is the saturated gain. ${L}$ is the cavity length. ${E}_{{\rm{c}}}$ is the energy of the “soliton crystal”

Based on these observations, Chen *et al* have derived a modified criterion for switching from QSML to CWML: ${\bar{g}}_{0}L{E}_{c}/{E}_{{\rm{G}}} \,>\, {q}_{0}{E}_{{\rm{a}}}/{E}_{{\rm{p}}}$ where ${E}_{{\rm{p}}}$ is the soliton energy, ${E}_{{\rm{G}}}$ and ${E}_{{\rm{a}}}$ are the saturation energies of the gain medium and the absorber, ${q}_{0}$ is the modulation depth of the saturable absorber, ${\bar{g}}_{0}$ is the saturated gain and $L$ the cavity length. ${E}_{{\rm{c}}}$ is the energy of the 'soliton crystal', which is typically tens to hundreds of times the single soliton energy. Compared to the conventional criterion ${E}_{{\rm{p}}}/{E}_{G}\, >\, {q}_{0}{E}_{{\rm{a}}}/{E}_{{\rm{p}}}$, the critical energy ${E}_{{\rm{p}}}$ predicted by this new expression is significantly reduced, because dynamic gain depletion counteracts relaxation oscillations in the pulse energy^3,6^.

Chen et al. also suggest that the rectangular bursts of gain-correlated QSML pulses may be regarded as 'quasi-single solitons' (QSS), lasting for *N* consecutive round-trips where $N=\sqrt{{\bar{g}}_{0}L{f}_{R}/{f}_{{\rm{c}}}}$, where ${f}_{R}$ and ${f}_{{\rm{c}}}$ are the cavity round-trip frequency and the repetition rate of the 'soliton crystal'. If the QSSs are viewed as single entities, the saturation energy is naturally lowered, while the critical single-pulse energy for CWML is dramatically reduced. Thus the general form of classical theory can be restored and a crossover between the two theories established.

This work is of particular importance in applications requiring a source of ultrashort pulses at multi-GHz repetition rates^7,8^ and is also relevant to ultrafast lasers built on other platforms^9^ and to the design of harmonically mode-locked lasers^10^. In identifying a new regime that favours CWML over QSML at multi-GHz repetition rates, the authors have added to our understanding of mode-locked laser systems.
